# dendextend: an R package for visualizing, adjusting and comparing trees of hierarchical clustering

**DOI:** 10.1093/bioinformatics/btv428

**Published:** 2015-07-23

**Authors:** Tal Galili

**Affiliations:** Department of Statistics and Operations Research, Tel Aviv University, Tel Aviv 6997801, Israel

## Abstract

**Summary:** *dendextend* is an R package for creating and comparing visually appealing tree diagrams. *dendextend* provides utility functions for manipulating dendrogram objects (their color, shape and content) as well as several advanced methods for comparing trees to one another (both statistically and visually). As such, *dendextend* offers a flexible framework for enhancing R's rich ecosystem of packages for performing hierarchical clustering of items.

**Availability and implementation:** The dendextend R package (including detailed introductory vignettes) is available under the GPL-2 Open Source license and is freely available to download from CRAN at: (http://cran.r-project.org/package=dendextend)

**Contact:** Tal.Galili@math.tau.ac.il

## 1 Introduction

Hierarchical cluster analysis (HCA) is a widely used family of unsupervised statistical methods for classifying a set of items into some hierarchy of clusters (groups) according to the similarities among the items. The R language (R Core Team, 2014)—a leading, cross-platform and open source statistical programming environment—has many implementations of HCA algorithms (Chipman and Tibshirani, 2006; Hornik, 2014; Schmidtlein *etal.*, 2010; Witten and Tibshirani, 2010). The output of these various algorithms is stored in the hclust object class, while the dendrogram class is an alternative object class that is often used as the go-to intermediate representation step for visualizing an HCA output.

In many R packages, a figure output is adjusted by supplying the plot function with both an object to be plotted and various graphical parameters to be modified (colors, sizes, etc.). However, different behavior happens in the (base R) plot.dendrogram function, in which the function is given a dendrogram object that contains within itself (most of) the graphical parameters to be used when plotting the tree. Internally, the dendrogram class is represented as a nested list of lists with attributes for colors, height, etc. (with useful methods from the *stats* package). Until now, no comprehensive framework has been available in R for flexibly controlling the various attributes in dendrogram's class objects.

The *dendextend* package aims to fill this gap by providing a significant number of new functions for controlling a dendrogram's structure and graphical attributes. It also implements methods for visually and statistically comparing different dendrogram objects. The package is extensively validated through unit-testing (Wickham, 2011), offers a C++ speed-up (Eddelbuettel and François, 2011) for some of the core functions through the *dendextendRcpp* package, and includes three detailed vignettes.

The *dendextend* package is primarily geared towards HCA. For phylogeny analysis, the phylo object class (from the *ape* package) is recommended (Paradis *etal.*, 2004). A comprehensive comparison of *dendextend, ape,* as well as other software for tree analysis, is available in the supplementary materials.

## 2 Description

### 2.1 Updating a dendrogram for visualization

The function set(*dend*, *what*, *value*), in *dendextend*, accepts a dendrogram (i.e. *dend*) as input and returns it after some adjustment. The parameter *what* is a character indicating the property of the tree to be adjusted (see Table 1) based on *value.* The user can repeatedly funnel a tree, through different configuration of the set function, until a desired outcome is reached.

**Table 1.** Available options for the ‘*what*’ parameter when using the set function for adjusting the look of a dendrogram

Description	Option name
Set the labels' names, color (per color, or with k clusters), size, turn to character	labels, labels_to_character, labels_colors, labels_cex, labels_to_character
Set the leaves' point type, color, size, height	leaves_pch, leaves_col, leaves_cex, hang_leaves
Set all nodes' point type, color, size	nodes_pch, nodes_col, nodes_cex
Set branches' line type, color, width - per branch, based on clustering the labels, and for specific labels	branches_lty, branches_col, branches_lwd, branches_k_color, by_labels_branches_lty, by_labels_branches_col, by_labels_branches_lwd

**Fig. 1.**
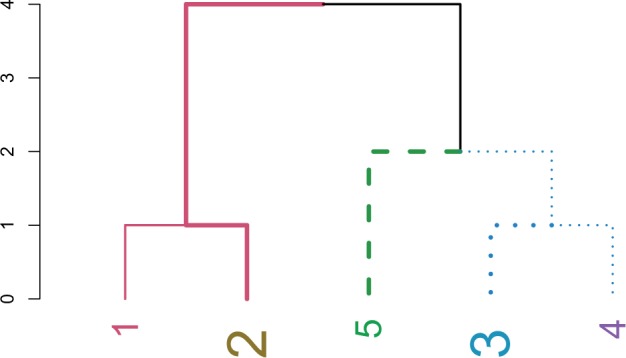
A dendrogram after modifying various graphical attributes

Figure 1 is created by clustering a vector of 1 to 5 into a dendrogram:

dend0 <−1:5%>%dist%>%hclust%>%as.dendrogram

The above code uses the convenient forward-pipe operator %>% (Milton and Wickham, 2014), which is just like running:

dend0 <−as.dendrogram(hclust(dist(1:5)))

Next, the tree is plotted after repeatedly using the set function:

$$\begin{array}{l} \mathrm{dend}0\%>\%\mathrm{set}\left(“\mathrm{labels}\_\mathrm{color}”\right)\;\%>\%\\ \mathrm{set}\left(“\mathrm{labels}\_\mathrm{cex}”,c\left(2,3\right)\right)\;\%>\%\\ \begin{array}{l} \mathrm{set}\left(“\mathrm{branches}\_\mathrm{lwd}”,c\left(2,4\right)\right)\;\%>\%\;\\ \mathrm{set}\left(“\mathrm{branches}\_k\_\mathrm{lty}”,k=3\right)\;\%>\% \end{array}\\ \mathrm{set}\left(“\mathrm{branches}\_k\_\mathrm{color}”,k=3\right)\%>\%\mathrm{plot} \end{array}$$

The ‘*value*’ vector is recycled in a depth-first fashion, with the root node considered as having a branch (which is not plotted by default). The parameters of the new tree can be explored using the functions get_nodes_attr and get_leaves_attr. Also, we can rotate and prune a tree with the respective functions.

### 2.2 Comparing two dendrograms

**Fig. 2.**
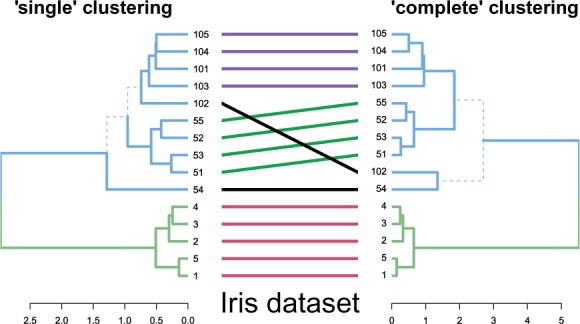
A tanglegram for comparing two clustering algorithms used on 15 flowers from the Iris dataset. Similar sub-trees are connected by lines of the same color, while branches leading to distinct sub-trees are marked by a dashed line

The tanglegram function allows the visual comparison of two dendrograms, from different algorithms or experiments, by facing them one in front of the other and connecting their labels with lines. Distinct branches are marked with a dashed line. For easier and nicer plotting, dendlist concatenates the two dendrograms together, while untangle attempts to rotate trees with un-aligned labels in search for a good layout. Figure 2 demonstrates a comparison of two clustering algorithms (single versus complete linkage) on a subset of 15 flowers from the famous Iris dataset. The entanglement function measures the quality of the tanglegram layout. Measuring the correlation between tree topologies can be calculated using different measures with cor.dendlist (Sokal and Rohlf, 1962), Bk_plot (Fowlkes and Mallows, 1983), or dist.dendlist. Permutation test and bootstrap confidence intervals are available. The above methods offer sensitivity and replicability analysis for researchers who are interested in validating their hierarchical clustering results.

## 3 Enhancing other packages

The R ecosystem is abundant with functions that use dendrograms, and *dendextend* offers many functions for interacting and enhancing their visual display: The function rotate_DendSer (Hurley and Earle, 2013) rotates a dendrogram to optimize a visualization-based cost function. Other functions allow the highlighting of un-even creation of clusters with the *dynamicTreeCut* package (Langfelder *etal.*, 2008), as well as of ‘significant’ clusters based on the *pvclust* package (Suzuki and Shimodaira, 2006). Previously mentioned functions can be combined to create a highly customized (rotated, colorful, etc.) static heatmap using heatplot.2 from *gplots* (Warnes *etal.*, 2014), or a D3 interactive heatmap using the *d3heatmap* package. The circlize_dendrogram function produces a simple circular tree layout, while more complex circular layouts can be achieved using the *circlize* package (Gu *etal.*, 2014). Aside from R base graphics, a *ggplot2* dendrogram may be created using the as.ggdend function.

*In conclusion,* the *dendextend* package simplifies the creation, comparison and integration of dendrograms into fine-tuned (publication quality) graphs. A demonstration of the package on various datasets is available in the supplementary materials.

## Supplementary Material

Supplementary Data
