# Supplementary material for: dendextend: an R package for visualizing, adjusting and comparing trees of hierarchical clustering
Source: Bioinformatics. 2015 Jul 23;31(22):3718–20. doi: 10.1093/bioinformatics/btv428 (PMC4817050; doi:10.1093/bioinformatics/btv428)
Supplement: Supplementary Data [file supp_31_22_3718__index.html]

dendextend: an R package for visualizing, adjusting and comparing trees of hierarchical clustering — Supplementary Data 

# dendextend: an R package for visualizing, adjusting and comparing trees of hierarchical clustering

## Supplementary Data

files

- Supplementary Data - zip file
