# Supplementary material for: dendextend: an R package for visualizing, adjusting and comparing trees of hierarchical clustering
Source: Bioinformatics. 2015 Jul 23;31(22):3718–20. doi: 10.1093/bioinformatics/btv428 (PMC4817050; doi:10.1093/bioinformatics/btv428)
Supplement: Supplementary Data [file supp_btv428_suppl_data.zip › Galili_dendextend_SM_2015-07-17.pdf]

Contents

Comparing *dendextend* with other R packages 2
dendextend's role in the ecosystem of R packages 2
A note on dendrogram/hclust/phylo object classes 3
Comparing features between *dendextend* and *ape(+other) packages* 3
Reading various tree formats into R 8
Graphical engines, circular layout, and interactivity 8
Untangle dendrograms 14
Other R packages and notes 15
Comparing R packages with other software 17
Summary 17
R and graphical-user-interfaces 17
Resources for comparing tree visualization software 17
Points per software 18
Table of comparisons 19
Fully commented R code for producing figure 2 of the paper 21

Comparing *dendextend* with other R packages

The *dendextend* package deals with manipulation, visualization and comparison of dendrogram objects in R. In this section we shall review existing R packages with similar or related features.

dendextend's role in the ecosystem of R packages

The *dendextend* R package should <u>not</u> be viewed solely as a stand-alone software. While it is useful for coloring branches and leaves, comparing trees and so on – it is also a necessary infrastructure that enables the communication between (and the combination of) other R packages. By adding new features to the `dendrogram` object class in R, *dendextend* allows a new level of flexibility in combining and leveraging existing packages within R.

To give just one <u>example</u>: a user can now import a phylogeny tree into R (using [*ape*]), convert that tree into a dendrogram object (with *dendextend*+[*ape*]), find an "optimal" rotation of the tree (using *dendextend+DendSer* packages), color the branches with different heights of the clusters (*dendextend*+[*dynamicTreeCut*]), then bold significant branches (*dendextend*+[*pvclust*]) and overlay this on top of a heatmap (*dendextend+gplots*), or with a *ggplot2* layout (*dendextend+ggplot2*). All of these steps can easily be combined using the pipe `%>%` operator (imported from the *magrittr* package, mentioned in the paper). These combinations require the various functions supplied by *dendextend*.

I demonstrate each of these steps in the other HTML material, as well as in the chapter "Enhancing other packages" in the *dendextend* tutorial, available in the following link:

http://cran.r-project.org/web/packages/dendextend/vignettes/introduction.html

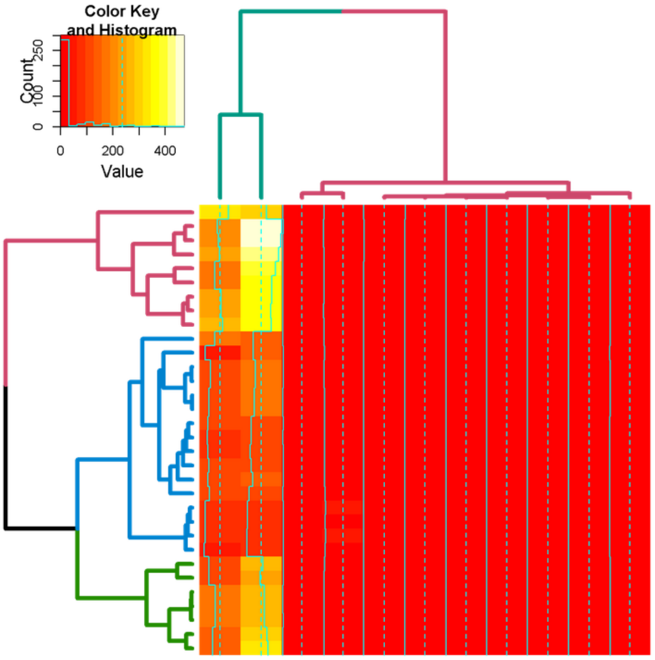

Fig 1. An example heatmap plot produced using *dendextend+DendSer+gplots*.

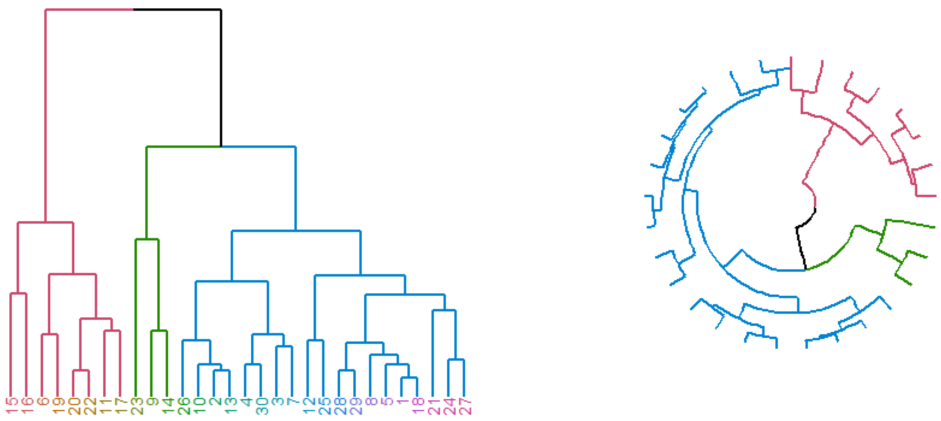

Fig 2. An example of a fan plot produced using *dendextend+ggplot2*.

While *dendextend* can be used for phylogeny analysis, I would strongly advise to also look into solutions for the `phylo` object type (from the *ape* package) since the tools for phylogeny research are very developed there. Nevertheless, dendrogram objects still play an important role in general hierarchical clustering (HC) models. The main benefit of *dendextend* is expected to come when enhancing other R packages that use hierarchical clustering.

A note on dendrogram/hclust/phylo object classes

In the R ecosystem there are three main classes of object for storing tree-like structures: `hclust`, `dendrogram`, and `phylo`. The first two were available since 2000, and `phylo` was introduced in 2004. Since then, there has been a simultaneous growth in the usage of all three object classes (as is evident in packages, books, and various online tutorials). Each of the object classes can be changed from one to the other using the functions: `as.dendrogram, as.hclust, as.phylo`. However, an important difference between `hclust`/`phylo` and `dendrogram` is that the latter contains its graphical parameters inside the object itself, while when plotting `hclust`/`phylo` we have to set these inside the plotting function. This means that if we have a package which relies on a dendrogram (for example, `heatmap.2` in the *gplot* package), then being able to control the color of branches when plotting the `phylo` object, will <u>not</u> help us to transfer these elements to the dendrogram based object. Hence, it was important to have these features re-implemented in *dendextend*.

As you shall see next, some (though not all) of the features that *dendextend* introduced to the `dendrogram` object class have already been around for either `hclust` or `phylo`. However, by bringing them to `dendrogram` objects, *dendextend* offers a much stronger connectivity between works done by various authors in different R packages that relies on the dendrogram object class.

Comparing features between *dendextend* and *ape(+other) packages*

Performing the tasks of manipulation, visualization and comparison of tree objects is possible thanks to many different packages. The *dendextend* package brings various capabilities to the dendrogram object class in base R, while *ape* (+others such as *phytools*,

phyloch, distory, phangorn, geiger) offers them for the `phylo` class (and other similar classes, such as `phylo4, phylog, phylog4`, etc.). Some features are common to different packages and for the sake of brevity their exact nuances are omitted.

The following table compares features available for both object classes.

Topic	Task	*dendextend* package (functions for `dendrogram` class)	*ape* package (functions for `phylo` class)<br><br>(When other R packages are used, they are mentioned explicitly)
Input	reading various formats of trees	See details below	
	A list of trees	`dendlist`	`multiPhylo`
Tree manipulation / visualization	See (change) labels<br><br>See attributes of nodes/leaves (size, height, color, width, type, etc…)	`Labels (Labels<-nleaves)`<br>`nnodes`<br>`get_nodes_attr`<br>`get_leaves_attr`<br>`get_branches_heights`<br>`get_childrens_heights`	Direct access through $:<br>$edge.length<br>$tip.label<br><br>`cophenetic`<br>`dist.nodes`<br><br>`tips`<br>(from *Geiger*)<br><br>`nodeId`<br>`edgeId`<br>(from *phylobase*)
	Describing relationships among phylogenetic nodes (add lines, node text, etc.)	`which_node`<br>`get_nodes_xy`<br>`noded_with_condition`	`which.edge`<br>`mrca`<br><br>`branching.times`<br><br>`ancestors`<br>`ancestor`<br>`siblings`<br>`children`<br>`descendants`<br>`MRCA`<br>`shortestPath`<br>`sumEdgeLength`<br>(from *phylobase*)
	Change nodes/leaves attributes (height, color, width, type, etc…)	**`set`** (a main gateway to most of the relevant options)<br><br>`assign_values_to_leaves_nodePar`<br>`assign_values_to_leaves_edgePar`<br>`assign_values_to_nodes_nodePar`<br>`assign_values_to_branches_edgePar`<br>`remove_branches_edgePar`<br>`remove_leaves_nodePar`<br>`branches_attr_by_clusters`<br>`branches_attr_by_labels`	`compute.brlen`<br><br>`rescale`<br>(from *Geiger*)<br><br>`multi2di` (branch collapse)

		raise.dendrogram<br>rank_branches<br>hang.dendrogram<br>collapse_branch<br><br>(note that dendrogram also holds graphical parameters of the tree, which is not the case for phylo objects.)	
	Branch length scaling using phylogeny analysis methods		Branch length scaling using ACDC; Pagel's (1999) lambda, delta and kappa parameters; and the Ornstein-Uhlenbeck alpha parameter (for ultrametric trees only) are available in *geiger*. *phytools* also allows branch length scaling.<br>Grafen's method can be done using *ape*.
	coloring leaves/branches (specialized functions)	set<br>color_branches<br>color_labels<br>labels_colors<br>(combined with various other functions from above)	User trees can be plotted using *ape*, *adephylo*, *phylobase*, *phytools* (see contMap), *ouch*, and *dendextend*; several of these have options for branch or taxon coloring based on some criterion (ancestral state, tree structure, etc.)<br>From tip.color/edge.color in plot.phylo to more complex control in ggtree.
	Prune labels (tips), various merging, re-branching and getting a sub-tree.	prune<br>unbranch<br>intersect_trees<br>flatten.dendrogram	drop.tip<br>root<br>unroot<br>multi2di<br>di2multi<br>node.leaves<br><br>drop.extinct<br>drop.random<br>subset.phylo<br>(from *Geiger*)<br><br>extractTree<br>(from *phylobase*)

			getNode<br>getEdge<br>(from [phylobase])
	Branch rotation	Rotate<br>sort.dendrogram<br>click_rotate<br>ladderize	Rotate<br>ladderize<br><br>Tree rearrangements (NNI and SPR) can be performed with [phangorn]
	Tree merging	merge.dendrogram (from *stats* in base R)	bind.tree
	Extract clusters	cutree.dendrogram	
	Add rectangles per cluster	rect.dendrogram	
	Color strips	colored_bars	
	Plotting functions/engines<br><br>(see section below regarding graphical engines)	plot.dendrogram (from base R)<br><br>ggplot.dendrogram (integration with *ggplot2*)<br><br>Alternative layouts are possible for ggplot.<br>For example: using +coord_polar(theta="x") will give a radial plot of the dendrogram. Please see notes below.	plot.phylo<br>(includes various layout options)<br><br>The packages: *ggphylo* *ggtree*
Comparing trees – visually	tanglegram plot	tanglegram	cophyloplot
	tanglegram plot – finding a good layout	untangle<br>(includes several methods: forward search, random search, two and one sided search)<br><br>entanglement	
	Bk plot	Bk_plot	
	plot a tree and on top of it the difference it has from another tree	dend_diff<br>(automatically used in tanglegram plots)	compare.phylo<br>(the [phyloch] package)<br><br>phylo.diff<br>(the [*distory*] package)
Comparing trees – statistically	checking if the two objects are equal (in labels, edge topology, and branchs' heights)	all.equal.dendrogram	all.equal.phylo
	find the edges that are present in the first tree but not in the second	distinct_edges	distinct.edges<br>(the [*distory*] package)

	geodesic distance		dist.multiPhylo (the *distory* package)
	Robinson-Foulds distance (a.k.a: symmetric difference, topological distance) i.e.: Sum of edges in both trees with labels that exist in only one of the two trees)	dist.dendlist	dist.topo (definitions seems slightly different)<br><br>dist.multiPhylo (the *distory* package)<br><br>treedist (the *phangorn* package)
	branch length score		dist.topo<br><br>treedist (the *phangorn* package)
	Path difference (yes/no weighted)		treedist (the *phangorn* package)
	Baker's gamma	cor_bakers_gamma	
	Cophenetic correlation	cor_cophenetic (see also cor.dendlist for creating a cor matrix)	
	Fowlkes-Mallows index	Bk<br>FM_index<br>(includes the theoretical mean and variance under the null hypotheses)	
Comparing trees – hypothesis tests and confidence intervals	Creating bootstrap samples of the tree (with replaced / repeated labels)	sample.dendrogram	rtree<br>rmtree<br>rcoal<br>boot.phylo<br><br>sim.bdtree (from *Geiger*)<br><br>Related functions are also in the packages:<br>*TreeSim*<br>*TESS*<br>*paleotree*
	Others		SH.test runs the Shimodaira--Hasegawa test (the *phangorn* package)<br><br>*kdetrees* implements a non-parametric method for identifying potential outlying observations in a collection of phylogenetic trees

			Rphylip provides an R interface for the PHYLIP package<br><br>Consensus trees are available in ape

Reading various tree formats into R

For reading tree formats into R (and *dendextend*), one should also use the *ape* package with functions such as `read.tree` (newick), `read.nexus` (or `readNexus` in *phylobase*), `read.dna` (for PHYLIP format), `read.cac` (for trees from the CAIC software), etc. to read a tree file and store it as a `phylo` object in R. Once in R, the *dendextend* package comes with a dedicated `as.dendrogram` function (i.e.: an S3 method `as.dendrogram.phylo`) for converting a `phylo` object into a `dendrogram` object.

The work process would be something like this:

```
library(ape)
tree_Newick <- "owls(((S:4.2,A:4.2):3.1,N:7.3):6.3,T:13.5);"
tree_phylo <- read.tree(text = tree_Newick)
library(dendextend)
tree_dend <- as.dendrogram(tree_phylo)
# tree_dend can now be used by dendextend
# or in a single line when using pipes:
tree_dend <- tree_Newick %>% read.tree(text = .) %>% as.dendrogram
```

There are many other ways to import trees into R (and then use `as.dendrogram` to make them malleable by *dendextend*): *treebase* can search for and load trees from the online tree repository TreeBASE, *rdryad* can pull data from the online data repository Dryad. *RNeXML* can read, write, and process metadata for the NeXML format. *PHYLOCH* can load trees from BEAST, MrBayes, and other phylogenetics programs (PHYLOCH is only available from the author's website).

Note that *dendextend* does not deal with the creation of trees. So hierarchical clustering of items, or reconstructing phylogenies (with distance based methods, maximum parsimony, maximum likelihood or any other method) – are not part of the scope of the package. To read more about R package which deals with that part, please look at "Phylogenetic Inference" in CRAN Task View: Phylogenetics

Graphical engines, circular layout, and interactivity

In general, *dendextend* offers the tools to prepare the tree for plotting. The actual plotting is done by the graphic engine. R has two main ones, and it is possible to transfer the tree to one of the two, depending on the needs. Starting from *dendextend* 0.18.0, there is an

integration with the *ape* package as well as with *ggplot2* package (which relies on the *grid* graphical engine).

The *dendextend* package comes with a dedicated `as.phylo` function (i.e.: an S3 method `as.phylo.dendrogram`) for converting a `dendrogram` object into a `phylo` object. This means that one can manipulate a `dendrogram` object in *dendextend*, turn it into a `phylo` object and then plot it using plotting functions from the *ape* package. Here is an example code+output for a radial plot using *ape*:

```
library(dendextend)
library(ape)
dend <- iris[1:30,-5] %>% dist %>% hclust %>% as.dendrogram
dend2 <- as.phylo(dend)
plot(dend2, type = "fan")
```

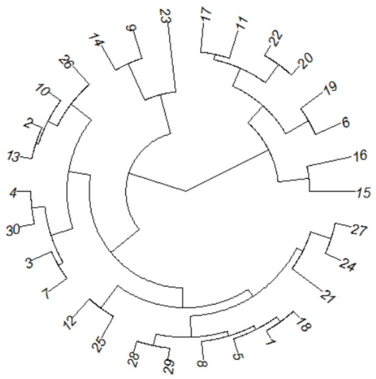


```
plot(dend2, type = "unrooted")
```

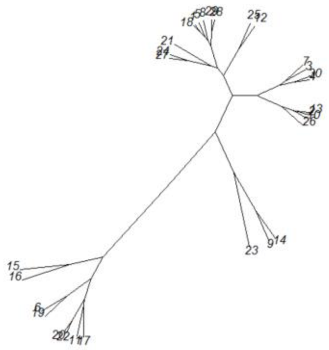

However, when going through the `phylo` object class, it is <u>not</u> possible to get the same level of control over the graphical parameters as with *dendextend.*

We may create a more (overly) complex example of a circular tree layout by using the *ggplot2* integration:

```
library(dendextend)
# Create a complex dend:
dend1 <- iris[1:30,-5] %>% dist %>% hclust %>% as.dendrogram %>%
   set("branches_k_color", k=3) %>% set("labels_colors")
# Create an even more complex dend:
dend2 <- dend1 %>%
   set("branches_lwd", c(1.5,1,1.5)) %>%
   set("branches_lty", c(1:4)) %>% set("labels_cex", c(.9,1.2))

ggd1 <- as.ggdend(dend1)
ggd2 <- as.ggdend(dend2)
```

```r
library(ggplot2)
library(gridExtra)
p1 <- ggplot(ggd1) # this is a new special "method" for ggdend objects
p2 <- ggplot(ggd1, labels = FALSE) + scale_y_reverse(expand = c(0.2, 0)) +
coord_polar(theta="x")

# Now let's plot it :)
grid.arrange(p1, p2, ncol=2)
```

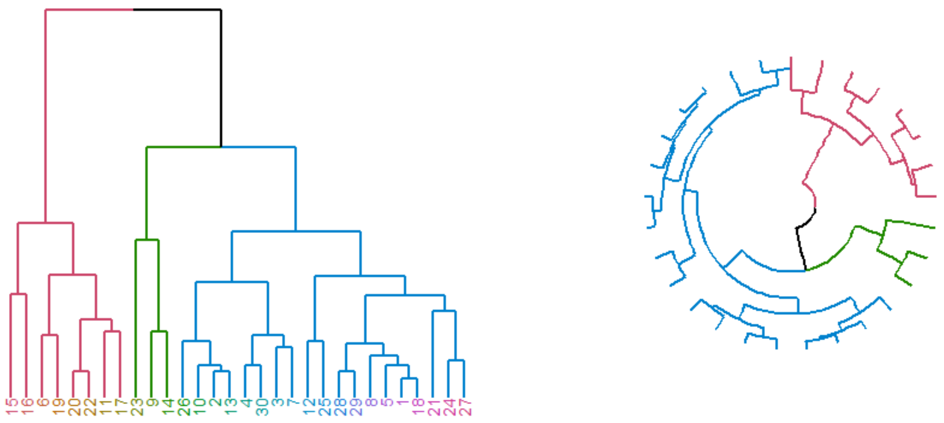

# plot the ***<u>more</u>*** *complex dend:*

```r
p1 <- ggplot(ggd2)
p2 <- ggplot(ggd2, labels = FALSE) + scale_y_reverse(expand = c(0.2, 0)) +
coord_polar(theta="x")
grid.arrange(p1, p2, ncol=2)
```

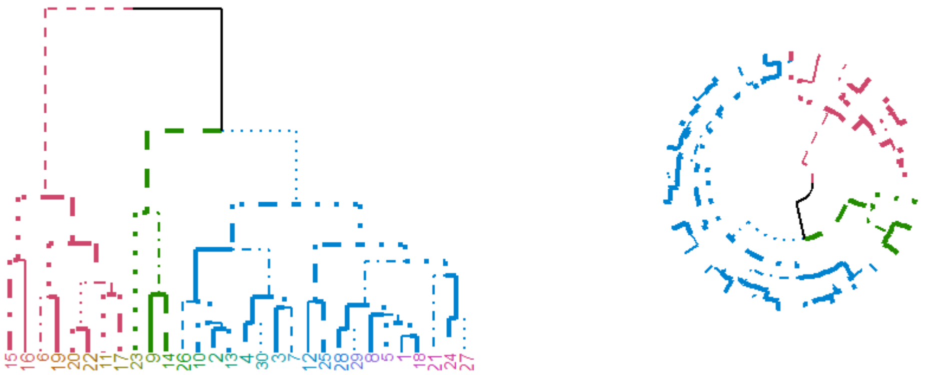

Another way of getting a basic circular tree layout is by using the `circlize_dendrogram` function, for example by running:

```r
circlize_dendrogram(dend2)
```

We will get:

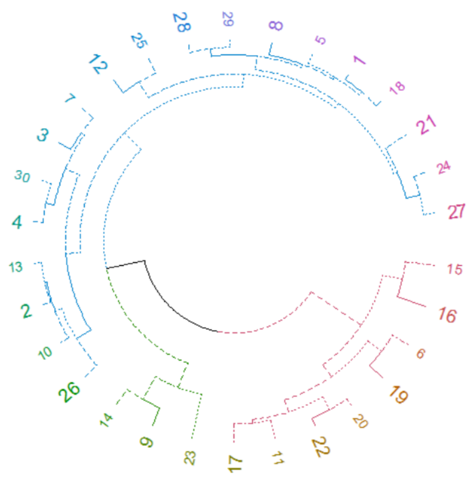

A simple circular tree layout

We may achieve more complex circular layouts by using the *circlize* package (note how these require many more lines of code), for example:

```
dend <- iris[1:40,-5] %>% dist %>% hclust %>% as.dendrogram %>%
   set("branches_k_color", k=3) %>% set("branches_lwd", c(5,2,1.5)) %>%
   set("branches_lty", c(1,1,3,1,1,2)) %>%
   set("labels_colors") %>% set("labels_cex", c(.9,1.2)) %>%
   set("nodes_pch", 19) %>% set("nodes_col", c("orange", "black", "plum",
NA))

library(circlize)

# In the following we get the dendrogram but can also get extra information
on top of it
circos.initialize("foo", xlim = c(0, 40))
circos.track(ylim = c(0, 1), panel.fun = function(x, y) {
   circos.rect(1:40-0.8, rep(0, 40), 1:40-0.2, runif(40), col =
rand_color(40), border = NA)
}, bg.border = NA)
circos.track(ylim = c(0, 1), panel.fun = function(x, y) {
   circos.text(1:40-0.5, rep(0, 40), labels(dend), col =
labels_colors(dend),
               facing = "clockwise", niceFacing = TRUE, adj = c(0, 0.5))
}, bg.border = NA, track.height = 0.1)
max_height = attr(dend, "height")
circos.track(ylim = c(0, max_height), panel.fun = function(x, y) {
   circos.dendrogram(dend, max_height = max_height)
}, track.height = 0.5, bg.border = NA)
circos.clear()
```

Will result in:

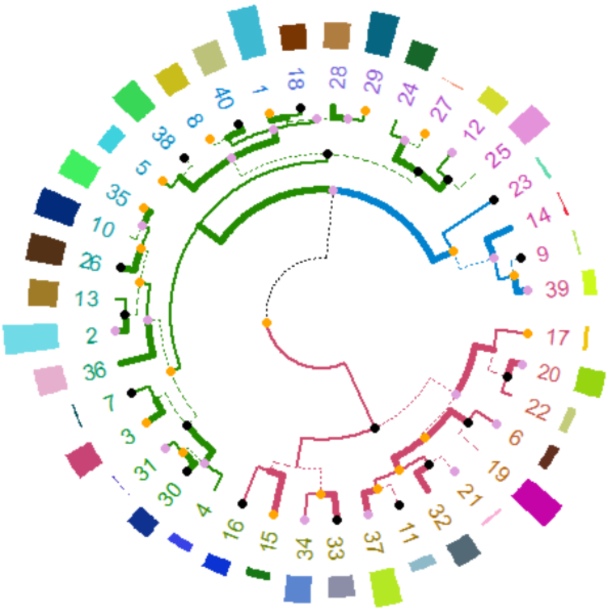

A complex circular tree layout

Regarding interactive visualization - the two basic graphical engines in R (*graphics* and *grid*), do not allow for fluid interactive visualization. While there are ways to bypass these restrictions in specific use cases (for example, see the `click_rotate` function in *dendextend,* or save a figure to pdf and zoom in/out in adobe reader), the current trend in the R world is to move to external devices (such as javascript based engines, as D3 and others), for allowing complex interactive visualization. *dendextend* offers some basic functionality in this direction (see the `d3dendrogram` function), but these functions are still in experimental beta-stage of development. The closest solution, currently, is exporting the plot to plot.ly, which offers some level of interaction (similar to what we get with iTol).

For the specific use-case of heatmaps, the new *d3heatmap* package (authored by Joe Cheng, and Tal Galili), allows for an interactive zoom-in, and hover-tooltip of a heatmap with (feature rich) dendrograms. For example, by running:

```
library(d3heatmap)
d3heatmap(mtcars, scale = "column", colors = "Blues", k_row = 4, k_col = 2)
```

We will get the following interactive heatmap (either in an html document, the RStudio viewer, or in a rmarkdown document):

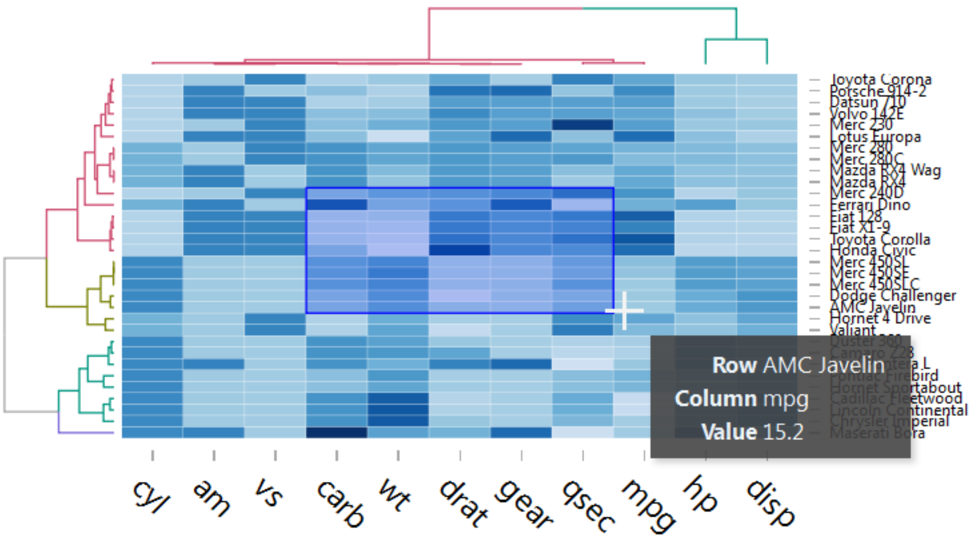

Interactive d3heatmap (relying also on dendextend)

Also, the *ggplot2* connection enables us to connect to more modern graphical engine. Specifically, the plot.ly web-based graphical engine. For example, by running the following code:

```
library(plotly)
set_credentials_file(...) # you'll need to get it from here:
https://plot.ly/ggplot2/getting-started/

ggplot(ggd1)
py <- plotly()
py$ggplotly()
```

Weget a tree plot, with some degree of interaction (zoom-in, manually changing plot parameters, etc.). You can see a live example here (the layout requires some more tweaking): https://plot.ly/~talgalili/6/y-vs-x/

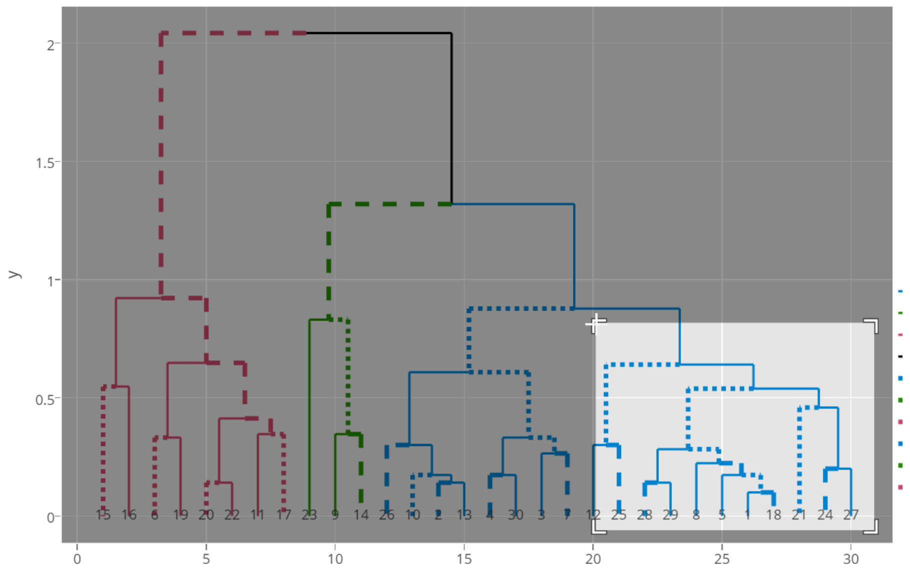

Zoom-in feature in a dendrogram's ggplot2 plot - exported to plot.ly

Untangle dendrograms

The `untangle` function in *dendextend* can be used on `dendlist` objects (which is a list of dendrograms), it rotates two trees in the `dendlist` so that their layout will have a lower `entanglement` measure when plotted as a `tanglegram`.

The `untangle` function has several possible methods, all use heuristics to optimize the tanglegram layout:

- "labels" – sorts the two dendrograms, as much as possible, based on the lexicographic ordering of the labels of the tree. If the two trees are identical, this will result in a tanglegram with perfectly parallel lines.
- "ladderize" - reorganizes the internal structure of the trees to get the ladderized effect when plotted (i.e.: that the smaller sub-trees are rotated to the right/top of the dendrogram).
- "random" – shuffles the two dendrograms R times (100 times by default), and measures the entanglement of each pairs of shuffled trees. The combination which minimizes the entanglement is returned.
- "step1side" - This is a greedy forward selection algorithm for rotating the tree and looking for a better match. It goes through rotating dend1 from top to bottom, each time keeping a rotation only if it offers an improvement over the previous layout. This is repeated until all one-step-look-ahead rotations are examined. This yields a local-optimal solution.
- "step2side" – similar to "step1side", one tree is held fixed and the other tree is rotated. This function goes through all of the k number of clusters (from 2 onward), and each time rotates the branch which was introduced in the new k'th cluster. This rotated tree is compared with the fixed tree, and if it has a better entanglement, it will be used for the following iterations. Once finished the rotated tree is held fixed, and the fixed tree is now rotated. This continues until a local optimal solution is reached.
- "DendSer" – rotates the two dendrograms based on their serration when using their cophenetic distance matrix with the `DendSer` function.

The general problem of tanglegram layout is NP-hard, and several papers were written to propose solutions. These algorithms have not (as of yet) been implemented in *dendextend*, yet some references might serve to show what progress might be made in the future:

- Böcker, S., Hüffner, F., Truss, A., & Wahlström, M. (2009). A faster fixed-parameter approach to drawing binary tanglegrams. *Lecture Notes in Computer Science (including Subseries Lecture Notes in Artificial Intelligence and Lecture Notes in Bioinformatics)*, *5917 LNCS*, 38–49. doi:10.1007/978-3-642-11269-0_3
- Buchin, K., Buchin, M., Byrka, J., & Nöllenburg, M. (2009). Drawing (complete) binary tanglegrams. *Graph Drawing*, (642), 1–12. doi:10.1007/978-3-642-00219-9_32

- Nöllenburg, M., Holten, D., Völker, M., & Wolff, A. (2008). Drawing Binary Tanglegrams: An Experimental Evaluation, 106–119. Retrieved from http://arxiv.org/abs/0806.0928
- Lozano, A., Pinter, R. Y., Rokhlenko, O., Valiente, G., & Ziv-ukelson, M. (n.d.). Seeded Tree Alignment and Planar Tanglegram Layout, 1–12.
- Scornavacca, C., Zickmann, F., & Huson, D. H. (2011). Tanglegrams for rooted phylogenetic trees and networks. *Bioinformatics*, *27*(13), 248–256. doi:10.1093/bioinformatics/btr210

Other R packages and notes

Beyond the above comparisons, there are other packages that should be mentioned (most offer either complementary functions or utilities already available in the *dendextend*'s toolset):

- The *igraph* package has the Tilford-Rheingold algorithm implemented, which is useful for plotting larger trees.
- The *dendroextras* package offers several functions for coloring a tree's labels, leaves and branches. All of these features are incorporated in *dendextend*, and rely on *dendextend's* implementation of the `cutree` function, while the *dendroextras* functions rely on the `hclust` version of the `cutree` function. This means that *dendextend* offers a more versatile implementation of these functions (i.e.: they can perform also on trees that cannot be coerced into `hclust`).
- The *ggdendro* package offers a connection between dendrogram and *ggplot2*, but does not export graphical parameters (colors/types/width etc of labels, lines and nodes). Hence, the code was imported into dendextend and extended in order to provide more capabilities with dendrogram plotting. The *ggdendro* package is expected to retire in the future, with all of its features migrated to *dendextend*.
- The *pvclust* package can calculate and add a "p-value" text on top of an hclust plot. The *dendextend* extends this by offering the same for dendrogram plots, also allowing the manipulation of branchs' colors and width to reflect the significance of the branch.
- The *dynamicTreeCut* package finds new ways of cutting a dendrogram tree for clustering the items in its leaves, but provide no way for visualizing it on top of the dendrogram tree itself. Doing so is possible using *dendextend*.
- The `A2Rplot` function (the *A2R* package is not available on CRAN, but the R code is available online) allows to plot the `hclust` object with basic control over coloring leaves and line parameters (line type, width, color) of branches.
- The *profdpm* package offers a Fowlkes-Mallows index for vectors of clusters (not dendrograms specific)
- The *sparcl* package provides the `ColorDendrogram` function that allows to add some color to the branches of the terminal nodes (based on `hclust`)
- Other packages exists for visualizing decision trees such as: *rpart*, *rpart.plot*, *partykit* (for **ctree**), and others. They usually deal with branch width/color, labels on the nodes (for conditions), and information on the leaves (result, and maybe a plot of outcome distribution). These specific needs have not been explored yet in *dendextend*, although eventually it might get there. If to quote from the help page of the dendrogram class what the R core team wrote: *"Class "dendrogram" provides general functions for handling tree-like structures. It is intended as a replacement for similar functions in hierarchical clustering and classification/regression trees, such*

that all of these can use the same engine for plotting or cutting trees." . So we might get there later on…

Some other useful relevant references to consider are: (collected from a mailing thread by Alastair Potts)

- de Vienne D.M., Giraud, T. and Martin, O.C. 2007. A Congruence Index for Testing Topological Similarity between Trees. *Bioinformatics* **23** (23): 3119-3124.
- Kuhner, M. K. and Felsenstein, J. (1994) Simulation comparison of phylogeny algorithms under equal and unequal evolutionary rates. *Molecular Biology and Evolution*, **11**, 459–468.
- Penny, D. and Hendy, M. D. (1985) The use of tree comparison metrics. *Systemetic Zoology*, **34**, 75–82.
- Shimodaira, H. and Hasegawa, M. (1999) Multiple comparisons of log-likelihoods with applications to phylogenetic inference. *Molecular Biology and Evolution*, **16**, 1114–1116.
- Steel M. A. and Penny P. (1993) *Distributions of tree comparison metrics - some new results*, Syst. Biol.,42(2), 126-141

Some relevant online references:

- http://cran.r-project.org/web/views/Phylogenetics.html
- http://cran.r-project.org/web/packages/ape/ape.pdf
- http://cran.r-project.org/web/packages/phylobase/phylobase.pdf
- http://cran.r-project.org/web/packages/geiger/geiger.pdf
- http://cran.r-project.org/web/packages/distory/distory.pdf
- http://cran.r-project.org/web/packages/phangorn/phangorn.pdf
- http://cran.r-project.org/web/packages/profdpm/profdpm.pdf
- http://www.r-phylo.org/wiki/HowTo/DataTreeManipulation
- http://www.researchgate.net/post/Is_there_a_software_that_can_compare_two_phylogenetic_trees_to_each_othe_face_to_face_comparison
- https://stat.ethz.ch/pipermail/r-sig-phylo/2011-March/001061.html

Comparing R packages with other software

Summary

R cannot compete with other tree viewers in term of explorative interactive visualization and interaction with the tree. On the other hand, R provides a substantial holistic ecosystem of methodologies and technologies to create, modify, analyze and visualize trees. From the software surveyed, only Dendroscope provided with tools to compare trees.

In that ecosystem, *dendextend* fits as an important connection point for combining different tools and capabilities from various R packages.

R and graphical-user-interfaces

In this section we will compare *dendextend* (a package extension for R) to other package. The most distinct difference between the two is that R is a command-based programming environment, while other common software provide a graphical user interface (GUI). I would like to quote the answer from the *ape* project to the question: "Why don't you develop a GUI for ape?":

> *There are at least three good reasons:*
>
> - *Developing a GUI is a lot work, and its maintenance and evolution is far from being straightforward.*
> - *Writing scripts of commands helps the users to see the logical integration of their analyses.*
> - *Repeating commands is almost always needed.*

I would like to add to the second point and say that R facilitates reproducible research using literate programming; a document that is a combination of content and data analysis code. Using the *knitr* package (the next generation of *Sweave*) can be used to blend the subject matter and R code so that a single document defines the content and the algorithms. This is how I wrote the vignette for the *dendextend* package. Everything that is shown in this page:

http://cran.r-project.org/web/packages/dendextend/vignettes/introduction.html

Was produced automatically from compiling this document:

https://github.com/talgalili/dendextend/blob/master/vignettes/introduction.Rmd

Resources for comparing tree visualization software

Performing a comprehensive comparison of all software for tree visualization is no small undertaking. The Wikipedia article - List of phylogenetic tree visualization software: https://en.wikipedia.org/wiki/List_of_phylogenetic_tree_visualization_software mentions 18 online software and 15 software applications (last update: December 2014). Probably other software exists in lists generally dealing with phylogeny analysis. For example:

- The Wikipedia article - List of phylogenetics software:
  https://en.wikipedia.org/wiki/List_of_phylogenetics_software
  mentions 52 software packages (last update: October 2014)
- The site: http://evolution.genetics.washington.edu/phylip/software.html
  is by Joe Felsenstein, from the University of Washington, and mentions 392 phylogeny packages and 54 free web servers. (last update: June 2014)
- The site: http://bioinfo.unice.fr/biodiv/Tree_editors.html
  is by Richard Christen, it lists 109 applications (last update: July 2010)
- The CRAN Task View: Phylogenetics
  http://cran.r-project.org/web/views/Phylogenetics.html
  lists 69 R packages alone for phylogeny analysis (last update: December 2014)
- https://www.biostars.org/p/2438/ (2011)

In order to keep the scope reasonable, several popular packages were chosen for this survey. Here are their names, and the resources describing their capabilities:

- TreeView –
  - http://taxonomy.zoology.gla.ac.uk/rod/treeview/help/contents.html
  - http://taxonomy.zoology.gla.ac.uk/rod/treeview/treeview_manual.html
- iTOL –
  - http://itol.embl.de/
  - http://itol.embl.de/help/help.shtml
- HyperTree –
  - http://kinase.com/web/current/hypertree/
  - http://kinase.com/tools/HyperTree.html#features
  - http://bioinformatics.oxfordjournals.org/content/16/7/660.full.pdf+html
- TreeIllustrator
  - http://www.bioinformatics.be/geert/
  - http://www.bioinformatics.be/geert/screenshots.php
- MixtureTree
  - http://www.mixturetree.net/index.php?page=Download
  - http://www.biomedcentral.com/content/pdf/1471-2105-12-111.pdf
- FigTree
  - http://tree.bio.ed.ac.uk/software/figtree/
- Archaeopteryx
  - https://sites.google.com/site/cmzmasek/home/software/archaeopteryx
- Dendroscope
  - http://ab.inf.uni-tuebingen.de/software/dendroscope/
  - http://ab.inf.uni-tuebingen.de/data/software/dendroscope3/download/manual.pdf
  - http://bioinformatics.oxfordjournals.org/content/27/13/i248.abstract

Points per software

Notes:

- Some subjective liberty was taken for how to compare features which are not fully comparable

- Since *dendextend* should not be treated as a stand-alone software, it is given one column (*dendextend++*) for features achieved by combining *dendextend* with other R packages (such as *ape*, *ggplot2*, etc.). The comparison of *dendextend*'s specific features, and how they are combined with those in other R packages, have been discussed in previous sections.

I tried finding all the features for each package, I might have missed a few (my apologies upfront).

Some points per package:

- TreeView – (C++, 2001) minimal
- iTOL (Flash, 2011) – online, nice radial layout with high level of control, allows to combine extra visual/statistical information on the plot
- HyperTree – (Java, 2011) special "fish-eye" layout for exploring larger trees interactively
- TreeIllustrator - (Java, 2005) a more updated TreeView
- MixtureTree – (C++/Python, Linux only!, 2012) a software for constructing trees (not visualizing/comparing them)
- MixtureTree Annotator – (Java, 2012) An old version of figtree aimed at viewing MixtureTree.
- FigTree – (Java, 2014) a nice modern tree viewer.
- Archaeopteryx – (Jave, 2014) a nice modern tree viewer, considered to be able to handle larger trees
- Dendroscope – (2013) A viewer designed for huge trees. The selling point is its efficiency, considered better than both FigTree and Archaeopteryx. Nonetheless, it does not seem to aim to produce publication-ready figures. Offers very sophisticated toolset for comparing trees.

Table of comparisons

Feature		*dendextend++*	TreeView	iTol	HyperTree	TreeIllustrator	MixtureTree	MixtureTree Annotator	FigTree	Dendroscope
Last updated		*2015*	2001	2011	2011	2005	2012	2012	2014	2013

	Programming			For tree up load						
Interface	Graphical (GUI)									
Location	Desktop									
	Online									
Creating trees	Phylogenetic Inference									
Reading/writing	common tree files (newick, nexus, etc.)									
	XML							?	?	
	Access to online databases							?	?	?
Tree Editor	Move branch					?				
	Pruning					?				
	Collapse branch									
	Reroot					?				
	Rotate branches									
	Ladderize left/right ("order")					?		?	?	
	Adjust branch lengths					?				?
Display	Save figures									
	Various Tree layout (Radial, Slanted cladogram, Rectangular cladogram, Phylogram, etc.)									
	hyperbolic ('fish-eye') layout									
	Graphical parameters of the tree (color, width, type, orientation, of labels, nodes, branches)									
	Add graphical elements on the plot (lines between nodes, text, legend)									
	Add additional statistical visualization on the graph (boxplot, barchars, piecharts, color strips, etc.)									

Interactive visualization	Zoom-in									
	Search (Zoom) to a leaf									
Perform statistical simulations /hypothesis -testing										
Comparing Trees	Visually (tanglegram)									
	Statistically									
	Consensus trees									

Fully commented R code for producing figure 2 of the paper

This code:

```
library(dendextend)

# Code for figure 2
# Full:
data(iris); ss <- c(1:5, 51:55, 101:105) # load data
iris1 <- iris[ss,-5] %>% dist %>% hclust(method = "single") %>%
as.dendrogram # produce dendrogram 1
iris2 <- iris[ss,-5] %>% dist %>% hclust(method = "complete") %>%
as.dendrogram # produce dendrogram 2
cols <- colorspace::rainbow_hcl(6)[c(3,5)]
dendlist(iris1, iris2) %>% # create a dendlist object
   set("branches_k_color", k=2, value = cols) %>% # color the branches of
both trees by cut of k=3
   set("branches_lwd",3)%>% # bold the line width of both trees to be 3
   highlight_distinct_edges(value = 1, edgePar = "lwd") %>% # thin the line
width of branches which are "distinct" (i.e.: do not replicate between the
two trees)
   untangle(method = "step2side") %>% # untangle the trees to have a better
looking layout
   tanglegram (sub="Iris dataset", main_left = "'single' clustering",
main_right = "'complete' clustering") # produce the tanglegram

# barplot(1:10, col = colorspace::rainbow_hcl(6)[c(3,5)])
```

Will produce this figure: (notice the bolded branches are the ones that relate to a clade that is replicated in both trees)

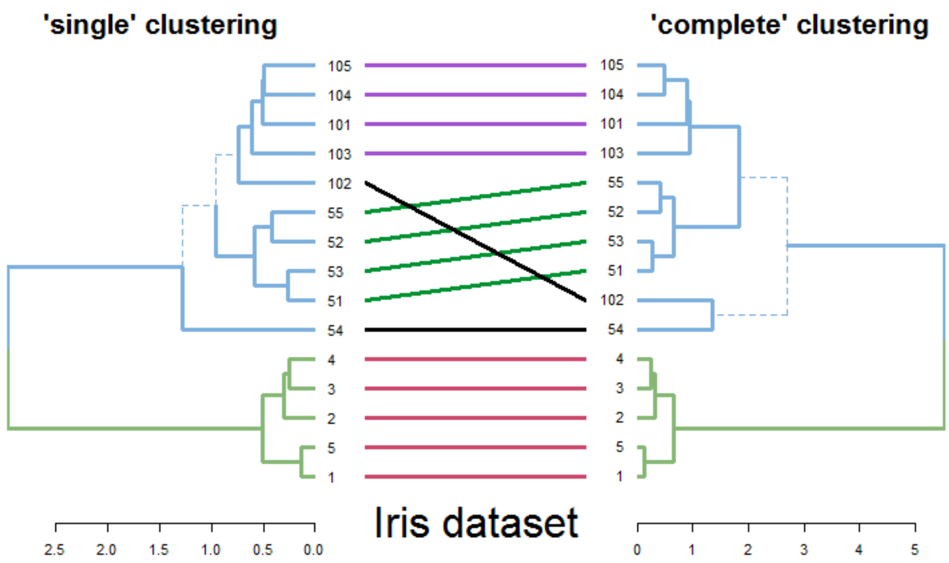
'single' clustering
'complete' clustering
105
104
101
103
102
55
52
53
51
54
4
3
2
5
1
105
104
101
103
55
52
53
51
102
54
4
3
2
5
1
Iris dataset
2.5
2.0
1.5
1.0
0.5
0.0
0
1
2
3
4
5
