# Supplementary material for: dendextend: an R package for visualizing, adjusting and comparing trees of hierarchical clustering
Source: Bioinformatics. 2015 Jul 23;31(22):3718–20. doi: 10.1093/bioinformatics/btv428 (PMC4817050; doi:10.1093/bioinformatics/btv428)
Supplement: Supplementary Data [file supp_btv428_suppl_data.zip › Hierarchical cluster analysis on famous data sets - enhanced with the dendextend package.html]

Hierarchical cluster analysis on famous data sets - enhanced with the dendextend package


# Hierarchical cluster analysis on famous data sets - enhanced with the *dendextend* package

#### *Tal Galili*

#### *2015-07-17*

- Introduction
- iris - Edgar Anderson’s Iris Data
  - Background
  - The 3 clusters from the “complete” method vs the real species category
  - Similarity/difference between various clustering algorithms
  - Clustering prediction of the 3 species classes
  - Conclusion
- khan - Microarray gene expression data set from Khan et al., 2001. Subset of 306 genes.
  - Background
  - Comparing the train vs test dendrograms
  - Conclusion
- votes.repub - Votes for Republican Candidate in Presidential Elections
  - Background
  - Heatmap
- animals - Attributes of Animals
  - Background
  - Heatmap

## Introduction

This document demonstrates, on several famous data sets, how the *dendextend* R package can be used to enhance Hierarchical Cluster Analysis (through better visualization and sensitivity analysis).

## iris - Edgar Anderson’s Iris Data

### Background

> The famous (Fisher’s or Anderson’s) iris data set gives the measurements in centimeters of the variables sepal length and width and petal length and width, respectively, for 50 flowers from each of 3 species of iris. The species are Iris setosa, versicolor, and virginica. (from `?iris`)

The Iris flower data set is fun for learning supervised classification algorithms, and is known as a difficult case for unsupervised learning. This is easily seen through the following Scatter Plot Matrix (SPLOM):

Define variables:

```
iris <- datasets::iris
iris2 <- iris[,-5]
species_labels <- iris[,5]
library(colorspace) # get nice colors
species_col <- rev(rainbow_hcl(3))[as.numeric(species_labels)]
```

SPLOM:

```
# Plot a SPLOM:
pairs(iris2, col = species_col,
      lower.panel = NULL,
       cex.labels=2, pch=19, cex = 1.2)

# Add a legend
par(xpd = TRUE)
legend(x = 0.05, y = 0.4, cex = 2,
   legend = as.character(levels(species_labels)),
    fill = unique(species_col))
par(xpd = NA)
```

We can see that the *Setosa* species are distinctly different from *Versicolor* and *Virginica* (they have lower petal length and width). But *Versicolor* and *Virginica* cannot easily be separated based on measurements of their sepal and petal width/length.

The same conclusion can be made by looking at the parallel coordinates plot of the data:

```
# http://blog.safaribooksonline.com/2014/03/31/mastering-parallel-coordinate-charts-r/
par(las = 1, mar = c(4.5, 3, 3, 2) + 0.1, cex = .8)
MASS::parcoord(iris2, col = species_col, var.label = TRUE, lwd = 2)

# Add Title
title("Parallel coordinates plot of the Iris data")
# Add a legend
par(xpd = TRUE)
legend(x = 1.75, y = -.25, cex = 1,
   legend = as.character(levels(species_labels)),
    fill = unique(species_col), horiz = TRUE)
```

```
par(xpd = NA)
```

### The 3 clusters from the “complete” method vs the real species category

The default hierarchical clustering method in `hclust` is “complete”. We can visualize the result of running it by turning the object to a dendrogram and making several adjustments to the object, such as: changing the labels, coloring the labels based on the real species category, and coloring the branches based on cutting the tree into three clusters.

```
d_iris <- dist(iris2) # method="man" # is a bit better
hc_iris <- hclust(d_iris, method = "complete")
iris_species <- rev(levels(iris[,5]))

library(dendextend)
dend <- as.dendrogram(hc_iris)
# order it the closest we can to the order of the observations:
dend <- rotate(dend, 1:150)

# Color the branches based on the clusters:
dend <- color_branches(dend, k=3) #, groupLabels=iris_species)

# Manually match the labels, as much as possible, to the real classification of the flowers:
labels_colors(dend) <-
   rainbow_hcl(3)[sort_levels_values(
      as.numeric(iris[,5])[order.dendrogram(dend)]
   )]

# We shall add the flower type to the labels:
labels(dend) <- paste(as.character(iris[,5])[order.dendrogram(dend)],
                           "(",labels(dend),")", 
                           sep = "")
# We hang the dendrogram a bit:
dend <- hang.dendrogram(dend,hang_height=0.1)
# reduce the size of the labels:
# dend <- assign_values_to_leaves_nodePar(dend, 0.5, "lab.cex")
dend <- set(dend, "labels_cex", 0.5)
# And plot:
par(mar = c(3,3,3,7))
plot(dend, 
     main = "Clustered Iris data set
     (the labels give the true flower species)", 
     horiz =  TRUE,  nodePar = list(cex = .007))
legend("topleft", legend = iris_species, fill = rainbow_hcl(3))
```

```
#### BTW, notice that:
# labels(hc_iris) # no labels, because "iris" has no row names
# is.integer(labels(dend)) # this could cause problems...
# is.character(labels(dend)) # labels are no longer "integer"
```

The same can be presented in a circular layout:

```
# Requires that the circlize package will be installed
par(mar = rep(0,4))
circlize_dendrogram(dend)
```

```
## Loading required namespace: circlize
```

These visualizations easily demonstrates how the separation of the hierarchical clustering is very good with the “Setosa” species, but misses in labeling many “Versicolor” species as “Virginica”.

The hanging of the tree also helps to locate extreme observations. For example, we can see that observation “virginica (107)” is not very similar to the Versicolor species, but still, it is among them. Also, “Versicolor (71)” is located too much “within” the group of Virginica flowers.

We can also explore the data using a heatmap. The rows are ordered based on the order of the hierarchical clustering (using the “complete” method). The colored bar indicates the species category each row belongs to. The color in the heatmap indicates the length of each measurement (from light yellow to dark red).

In the heatmap we also see how the Setosa species has low petal values (in light yellow), but it is very difficult to see any clear distinction between the other two species.

```
some_col_func <- function(n) rev(colorspace::heat_hcl(n, c = c(80, 30), l = c(30, 90), power = c(1/5, 1.5)))

# scaled_iris2 <- iris2 %>% as.matrix %>% scale
# library(gplots)
gplots::heatmap.2(as.matrix(iris2), 
          main = "Heatmap for the Iris data set",
          srtCol = 20,
          dendrogram = "row",
          Rowv = dend,
          Colv = "NA", # this to make sure the columns are not ordered
          trace="none",          
          margins =c(5,0.1),      
          key.xlab = "Cm",
          denscol = "grey",
          density.info = "density",
          RowSideColors = rev(labels_colors(dend)), # to add nice colored strips        
          col = some_col_func
         )
```

```
## NULL
```

We can get an interactive heatmap by using the `d3heatmap` package/function:

```
d3heatmap::d3heatmap(as.matrix(iris2),
          dendrogram = "row",
          Rowv = dend,
          colors = "Greens",
          # scale = "row",
          width = 600,
          show_grid = FALSE)
```

### Similarity/difference between various clustering algorithms

We may ask ourselves how many different results we could get if we would use different clustering algorithms (`hclust` has 8 different algorithms implemented). For the purpose of this analysis, we will create all 8 hclust objects, and chain them together into a single `dendlist` object (which, as the name implies, can hold a bunch of dendrograms together for the purpose of further analysis).

```
hclust_methods <- c("ward.D", "single", "complete", "average", "mcquitty", 
        "median", "centroid", "ward.D2")
iris_dendlist <- dendlist()
for(i in seq_along(hclust_methods)) {
   hc_iris <- hclust(d_iris, method = hclust_methods[i])   
   iris_dendlist <- dendlist(iris_dendlist, as.dendrogram(hc_iris))
}
names(iris_dendlist) <- hclust_methods
iris_dendlist
```

```
## $ward.D
## 'dendrogram' with 2 branches and 150 members total, at height 199.6205 
## 
## $single
## 'dendrogram' with 2 branches and 150 members total, at height 1.640122 
## 
## $complete
## 'dendrogram' with 2 branches and 150 members total, at height 7.085196 
## 
## $average
## 'dendrogram' with 2 branches and 150 members total, at height 4.062683 
## 
## $mcquitty
## 'dendrogram' with 2 branches and 150 members total, at height 4.497283 
## 
## $median
## 'dendrogram' with 2 branches and 150 members total, at height 2.82744 
## 
## $centroid
## 'dendrogram' with 2 branches and 150 members total, at height 2.994307 
## 
## $ward.D2
## 'dendrogram' with 2 branches and 150 members total, at height 32.44761 
## 
## attr(,"class")
## [1] "dendlist"
```

Next, we can look at the cophenetic correlation between each clustering result using `cor.dendlist`. (This can be nicely plotted using the `corrplot` function from the *corrplot* package):

```
iris_dendlist_cor <- cor.dendlist(iris_dendlist)
iris_dendlist_cor
```

```
##             ward.D    single  complete   average  mcquitty    median
## ward.D   1.0000000 0.9836838 0.5774013 0.9841333 0.9641103 0.9451815
## single   0.9836838 1.0000000 0.5665529 0.9681156 0.9329029 0.9444723
## complete 0.5774013 0.5665529 1.0000000 0.6195121 0.6107473 0.6889092
## average  0.9841333 0.9681156 0.6195121 1.0000000 0.9828015 0.9449422
## mcquitty 0.9641103 0.9329029 0.6107473 0.9828015 1.0000000 0.9203374
## median   0.9451815 0.9444723 0.6889092 0.9449422 0.9203374 1.0000000
## centroid 0.9809088 0.9903934 0.5870062 0.9801444 0.9499123 0.9403569
## ward.D2  0.9911648 0.9682507 0.6096286 0.9895131 0.9829977 0.9445832
##           centroid   ward.D2
## ward.D   0.9809088 0.9911648
## single   0.9903934 0.9682507
## complete 0.5870062 0.6096286
## average  0.9801444 0.9895131
## mcquitty 0.9499123 0.9829977
## median   0.9403569 0.9445832
## centroid 1.0000000 0.9737886
## ward.D2  0.9737886 1.0000000
```

```
corrplot::corrplot(iris_dendlist_cor, "pie", "lower")
```

From the above figure, we can easily see that most clustering methods yield very similar results, except for the complete method (the default method in `hclust`), which yields a correlation measure of around 0.6.

The default cophenetic correlation uses pearson’s measure, but what if we use the spearman’s correlation coefficient?

```
iris_dendlist_cor_spearman <- cor.dendlist(iris_dendlist, method_coef = "spearman")
corrplot::corrplot(iris_dendlist_cor_spearman, "pie", "lower")
```

We can see that the correlations are not so strong, indicating a behavior that is dependent on some items which are very distant from one another having an influence on the pearson’s correlation more than that of the spearman’s correlation.

To further explore the similarity and difference between the alternative clustering algorithms, we can turn to using the `tanglegram` function (which works for either two `dendrogram`s, or a `dendlist`).

First, let us see two methods which are very similar: ward.D vs ward.D2. From a first glance, we can see how they both give the same result for the top 3 clusters. However, since they are both ladderizes (i.e.: having their smaller branch rotated to be higher for each node), we can see that their clustering is not identical (due to the crossings).

```
# The `which` parameter allows us to pick the elements in the list to compare
iris_dendlist %>% dendlist(which = c(1,8)) %>% ladderize %>% 
   set("branches_k_color", k=3) %>% 
   # untangle(method = "step1side", k_seq = 3:20) %>%
   # set("clear_branches") %>% #otherwise the single lines are not black, since they retain the previous color from the branches_k_color.
   tanglegram(faster = TRUE) # (common_subtrees_color_branches = TRUE)
```

Next, let us look at two methods which also have a high cophenetic correlation: ward.D vs the average:

```
# The `which` parameter allows us to pick the elements in the list to compare
iris_dendlist %>% dendlist(which = c(1,4)) %>% ladderize %>% 
   set("branches_k_color", k=2) %>% 
   # untangle(method = "step1side", k_seq = 3:20) %>%
   tanglegram(faster = TRUE) # (common_subtrees_color_branches = TRUE)
```

We see that when it comes to the major clusters, the two algorithms perform quite similarly.

However, how are they doing inside each of the clusters? It is quite difficult to compare the two because of the high value in ward.D. For comparison purposes, we can “rank” the heights of the branches in the two dendrograms (while still preserving their internal order). Next, we can highlight the shared common sub-trees (with different colors), and the distinct edges (with a dashed line):

```
# The `which` parameter allows us to pick the elements in the list to compare
iris_dendlist %>% dendlist(which = c(1,4)) %>% ladderize %>% 
   # untangle(method = "step1side", k_seq = 3:20) %>%
   set("rank_branches") %>%
   tanglegram(common_subtrees_color_branches = TRUE)
```

We have 39 sub-trees that are identical between the two dendrograms:

```
length(unique(common_subtrees_clusters(iris_dendlist[[1]], iris_dendlist[[4]]))[-1])
```

```
## [1] 39
```

```
# -1 at the end is because we are ignoring the "0" subtree, which indicates leaves that are singletons.
```

What we can learn from this is that actually the two algorithms seem to give quite different results in the high resolution (higher cuts). However, since both capture the two major clusters (Setosa vs the others), they are considered quite similar by the cophenetic correlation.

But what about the “complete” method (that got a lower cophenetic correlation than the other methods)? When we compare “complete” vs “average”, we can quickly see that in the “complete” method, the splitting of the clusters is much more balanced, and mixes the “Setosa” species with another one. This is probably the cause for the big difference found in the cophenetic correlation between the “complete method” and the other clustering methods:

```
iris_dendlist %>% dendlist(which = c(3,4)) %>% ladderize %>% 
   untangle(method = "step1side", k_seq = 2:6) %>%
   set("branches_k_color", k=2) %>% 
   tanglegram(faster = TRUE) # (common_subtrees_color_branches = TRUE)
```

We can quickly plot all 8 methods to see this phenomenon (i.e.: that “complete” has its smaller cluster larger than it is in all the other clustering methods):

```
par(mfrow = c(4,2))
for(i in 1:8) {
   iris_dendlist[[i]] %>% set("branches_k_color", k=2) %>% plot(axes = FALSE, horiz = TRUE)
   title(names(iris_dendlist)[i])
}
```

It seems that the cophenetic correlation is very biased towards the influence of the main clusters. Another correlation measure to use is the `cor_common_nodes` correlation (giving the proportion of nodes which share the exact same list of labels in both dendrograms). We can also check it out:

```
iris_dendlist_cor2 <- cor.dendlist(iris_dendlist, method = "common")
iris_dendlist_cor2
```

```
##             ward.D    single  complete   average  mcquitty    median
## ward.D   1.0000000 0.7324415 0.8595318 0.8461538 0.8361204 0.7458194
## single   0.7324415 1.0000000 0.7324415 0.7491639 0.7458194 0.7591973
## complete 0.8595318 0.7324415 1.0000000 0.8060201 0.7993311 0.7491639
## average  0.8461538 0.7491639 0.8060201 1.0000000 0.8494983 0.7892977
## mcquitty 0.8361204 0.7458194 0.7993311 0.8494983 1.0000000 0.7859532
## median   0.7458194 0.7591973 0.7491639 0.7892977 0.7859532 1.0000000
## centroid 0.7324415 0.7625418 0.7290970 0.7725753 0.7759197 0.8528428
## ward.D2  0.8795987 0.7324415 0.8294314 0.8294314 0.8294314 0.7558528
##           centroid   ward.D2
## ward.D   0.7324415 0.8795987
## single   0.7625418 0.7324415
## complete 0.7290970 0.8294314
## average  0.7725753 0.8294314
## mcquitty 0.7759197 0.8294314
## median   0.8528428 0.7558528
## centroid 1.0000000 0.7357860
## ward.D2  0.7357860 1.0000000
```

And plot it:

```
corrplot::corrplot(iris_dendlist_cor2, "pie", "lower")
```

This gives us another perspective on our clustering algorithms. We can see that most methods have around 75% common nodes with one another. Centroid and median seem relatively close to one another, as well as ward.D2 and ward.D to one another and to complete, average, and mcquitty (as compared to the other methods).

### Clustering prediction of the 3 species classes

Lastly, we would like to see which of the different clustering algorithms came the closest to detecting the 3 flower species (when using a cut of k=3).

For this purpose, we compare the clustering solution of each algorithm with the real clusters, using the Fowlkes-Mallows Index (also using in the package for the `Bk_plot`). This measure is similar to rand (or rand adjusted) index, and gives a value of 1 when the two clusters confirm, and 0 when they do not.

```
get_ordered_3_clusters <- function(dend) {
   cutree(dend, k = 3)[order.dendrogram(dend)]
}

dend_3_clusters <- lapply(iris_dendlist, get_ordered_3_clusters)

compare_clusters_to_iris <- function(clus) {FM_index(clus, rep(1:3, each = 50), assume_sorted_vectors = TRUE)}

clusters_performance <- sapply(dend_3_clusters, compare_clusters_to_iris)
dotchart(sort(clusters_performance), xlim = c(0.7,1),
         xlab = "Fowlkes-Mallows Index (from 0 to 1)",
         main = "Perormance of clustering algorithms \n in detecting the 3 species",
         pch = 19)
```

We can see that the “median” method did the best, although similar results were achieved by ward.D2, average, ward.D, and mcquitty. However, the complete, centroid, and single method did worse in our case.

### Conclusion

The Iris data set is only 4-dimensional, making it possible to explore using pairs plot (SPLOM) or parallel coordinates plot. It is clear from these that two main clusters are visible, while the separation of the third cluster is difficult.

In the above analysis, we learned that the complete method fails to do the proper separation of the two main clusters when cut in k=2 (but succeeds in doing it, if moving to k=3 clusters). This is different from all the other 7 methods available in `hclust`, which do succeed in separating the 2 main clusters from the beginning (i.e.: for k=2).

We also noticed that all clustering algorithms share a relatively high proportion of common nodes (between 75% to 90%).

Lastly, when it came to trying to separating the flowers into 3 species, the median clustering method did the best, while the single method did the worst in this regard.

While the Iris data set is well known, I hope the above analysis was able to offer some new perspectives on the performance of the different hierarchical clustering methods.

## khan - Microarray gene expression data set from Khan et al., 2001. Subset of 306 genes.

### Background

> Khan contains gene expression profiles of four types of small, round, blue cell tumors of childhood (SRBCT) published by Khan et al. (2001). It also contains further gene annotation retrieved from SOURCE at http://source.stanford.edu/.

This interesting data set offers two interesting items:

- train: data.frame of 306 rows and 64 columns. The training data set of 64 arrays and 306 gene expression values
- test: data.frame, of 306 rows and 25 columns. The test data set of 25 arrays and 306 genes expression values

This way we can create a hierarchical clustering on the 306 genes expression values on the train and the test data and compare the two to see the stability of the results.

We define the variables:

```
train <- dendextend::khan$train
test <- dendextend::khan$test
```

And create the dendrograms:

```
d_train <- train %>% dist %>% hclust %>% as.dendrogram
d_test <- test %>% dist %>% hclust %>% as.dendrogram
d_train_test <- dendlist(train = d_train, test = d_test)
```

### Comparing the train vs test dendrograms

Using a cophenetic correlation, we can see the two trees have some similarity (0.57):

```
d_train_test %>% cor.dendlist
```

```
##           train      test
## train 1.0000000 0.5708019
## test  0.5708019 1.0000000
```

However, when looking at the cophenetic correlation with the spearman correlation coefficiant, the value is lower (0.49) indicating that some of the similarity is due to a small number of items, distant from the others, which are correlated similarly in the two trees:

```
d_train_test %>% cor.dendlist(method_coef = "spearman")
```

```
##           train      test
## train 1.0000000 0.4971936
## test  0.4971936 1.0000000
```

We may ask at which level of cutting the dendrogram we get the “best” level of similarity. For this we may turn to the Bk plot. The plots shows us that at around 7 clusters the groups in the two are starting to look significantly similar. (Note that significantly does not mean substantially)

```
Bk_plot(d_train, d_test, k = 2:30, xlim = c(2,30))
```

Next, we compare the results with a tanglegram. We make sure to color the connecting line with the colors of the branches of the train (left) dendrogram. This can help us see which patterns are somewhat preserved between the two trees.

```
pre_tang_d_train_test <- d_train_test %>% ladderize %>% # untangle %>%
   set("branches_k_color", k = 7)
train_branches_colors <- get_leaves_branches_col(pre_tang_d_train_test$train)
pre_tang_d_train_test %>% tanglegram(fast = TRUE, color_lines = train_branches_colors)
```

We can see that the top most (small) cluster is somewhat preserved between the two trees. However, a large spaghetti-like tangle of lines is indicating that the two trees are far from being identical.

If we look only at subtrees of the two dendrograms so that they include only genes that are clustered with genes in both trees, we get only 14 genes (while the original trees had 306 genes). We can see how we have several groups of pairs of genes, and one group with four genes clustered together in both trees:

```
# This was calculated before
# d_train_test_common <- d_train_test %>% prune_common_subtrees.dendlist
# d_train_test_common
d_train_test_common %>% untangle %>%  tanglegram(common_subtrees_color_branches = TRUE)
```

Trees’ sizes:

```
d_train_test %>% nleaves
```

```
## train  test 
##   306   306
```

```
d_train_test_common %>% nleaves
```

```
## train  test 
##    14    14
```

### Conclusion

To conclude: we see that the clustering algorithm resulted in trees which are significantly similar in both the training and the test data sets beyond chance, but that this similarity is restricted to only a very small proportion of genes.

## votes.repub - Votes for Republican Candidate in Presidential Elections

### Background

> This is a data frame with the percentage of votes given to the republican candidate in presidential elections from 1856 to 1976. Rows represent the 50 states, and columns the 31 elections.

> Source: S. Peterson (1973): A Statistical History of the American Presidential Elections. New York: Frederick Ungar Publishing Co. Data from 1964 to 1976 is from R. M. Scammon, American Votes 12, Congressional Quarterly.

Define variables:

```
votes.repub <- cluster::votes.repub
```

These data can be visualized using a (costumed made) parallel coordinates plot:

```
years <- as.numeric(gsub("X", "", colnames(votes.repub)))

par(las = 2, mar = c(4.5, 3, 3, 2) + 0.1, cex = .8)
# MASS::parcoord(votes.repub, var.label = FALSE, lwd = 1)
matplot(1L:ncol(votes.repub), t(votes.repub), type = "l", col = 1, lty = 1,
        axes = F, xlab = "", ylab = "")
axis(1, at = seq_along(years), labels = years)
axis(2)
# Add Title
title("Votes for Republican Candidate\n in Presidential Elections \n (each line is a country - over the years)")
```

### Heatmap

This is a nice example when the parallel coordinates plot has some serious limitations: it does not help us detect the states, we fail to see the missing value patterns, and it is tricky to see clusters in general (due to the large number of threads).

For these data, it can be quite helpful to see a heatmap of the votes across the years. The ordering of the rows is tricky. First, the distance of the vectors (later used for the clustering) should be done after transformation (since we are dealing with proportion of votes). In this case, I used the arcsin transformation (a logit transformation could also work, but the arcsin is safer for dealing with 0/1 observations). But given the clusters, we wish to order the leaves (as much as possible), in order to take into account the missing value clusterings. So we, in fact, have two clusters, one for the raw values, and another for the “shadow matrix” (i.e.: the matrix with 0/1, indicating if a value was missing or not).

```
arcsin_transformation <- function(x) asin(x/100)

dend_NA <- votes.repub %>% is.na %>%
   dist %>% hclust %>% as.dendrogram %>% ladderize

dend <- votes.repub %>% arcsin_transformation %>%
   dist %>% hclust(method = "com") %>% as.dendrogram %>%
   rotate(labels(dend_NA)) %>%
   color_branches(k=3)

# some_col_func <- function(n) rev(colorspace::heat_hcl(n, c = c(80, 30), l = c(30, 90), power = c(1/5, 1.5)))
some_col_func <- colorspace::diverge_hcl


# par(mar = c(3,3,3,3))
# library(gplots)
gplots::heatmap.2(as.matrix(votes.repub), 
          main = "Votes for\n Republican Presidential Candidate\n (clustered using complete)",
          srtCol = 60,
          dendrogram = "row",
          Rowv = dend,
          Colv = "NA", # this to make sure the columns are not ordered
          trace="none",          
          margins =c(3,6),      
          key.xlab = "% Votes for Republican\n Presidential Candidate",
          labCol = years,
          denscol = "grey",
          density.info = "density",
          col = some_col_func
         )
```

```
## NULL
```

```
          # RowSideColors = rev(labels_colors(dend)), # to add nice colored strips
```

How much of a difference would we get if we used another clustering algorithm?

We first calculate the clustering using 8 different methods:

```
hclust_methods <- c("ward.D", "single", "complete", "average", "mcquitty", 
        "median", "centroid", "ward.D2")
votes.repub_dendlist <- dendlist()

for(i in seq_along(hclust_methods)) {
   tmp_dend <- votes.repub %>% arcsin_transformation %>% dist %>% hclust(method = hclust_methods[i]) %>% as.dendrogram 
   votes.repub_dendlist <- dendlist(votes.repub_dendlist, tmp_dend)
}
names(votes.repub_dendlist) <- hclust_methods
# votes.repub_dendlist
```

Next, we can look at the cophenetic correlation between each clustering result using `cor.dendlist`. (This can be nicely plotted using the `corrplot` function from the *corrplot* package):

```
corrplot::corrplot(cor.dendlist(votes.repub_dendlist), "pie", "lower")
```

We see that the “complete” method is somewhat similar to the ward.D/ward.D2 methods, but there is less similarity with the other methods. We can see that the methods “average”, “mcquitty” and “median”, all give somewhat similar results. So by using “average”, we will see an alternative presentation that represents (in a sense) three other clustering solutions.

We can look at the heatmap of the “average” method. However, as you can see, it is not very helpful in seeing the difference between the two clustering solutions.

```
## NULL
```

Let’s look at the tanglegram of the two methods to get a better insight into the differences between the two:

```
dend_com <- votes.repub %>% arcsin_transformation %>%
   dist %>% hclust(method = "com") %>% as.dendrogram %>%
   rotate(labels(dend_NA)) %>%
   color_branches(k=3) # %>% ladderize
dend_ave <- votes.repub %>% arcsin_transformation %>%
   dist %>% hclust(method = "ave") %>% as.dendrogram %>%
   rotate(labels(dend_NA)) %>%
   color_branches(k=3) # %>% ladderize

# The orders were predefined after using untangle("step2side")
# They are omitted here to save running time.
dend_com <- rotate(dend_com, ord1)
dend_ave <- rotate(dend_ave, ord2)

dends <- dendlist(complete = dend_com, average = dend_ave) # %>% untangle("step2side")
dends  %>% tanglegram(margin_inner = 7)
```

We see that the two clusterings give similar results for: “Alabama”, “Georgia”, “Louisiana”, “Arkansas”, “Florida”, “Texas”, “South Carolina”, “Mississippi”.

There are also several other sub-trees which are identical between the two methods. The biggest difference lies in several “rouge” states that are placed differently in the two clustering algorithms. They are: Vermont, Michigan, Maine, Hawaii, New Jersey, West Virginia, and Oklahoma.

A better understanding of the data requires a much more in-depth historical perspective than is within the scope of the current analysis.

## animals - Attributes of Animals

### Background

> This data set considers 6 binary attributes for 20 animals.

> see Struyf, Hubert & Rousseeuw (1996), in agnes.

Define variables:

```
animals <- cluster::animals

colnames(animals) <- c("warm-blooded", 
                       "can fly",
                       "vertebrate",
                       "endangered",
                       "live in groups",
                       "have hair")
```

### Heatmap

This is a good example for using a heatmap + colored branches.

```
dend_r <- animals %>% dist(method = "man") %>% hclust(method = "ward.D") %>% as.dendrogram %>% ladderize %>%
    color_branches(k=4)

dend_c <- t(animals) %>% dist(method = "man") %>% hclust(method = "com") %>% as.dendrogram %>% ladderize%>%
    color_branches(k=3)


# some_col_func <- function(n) rev(colorspace::heat_hcl(n, c = c(80, 30), l = c(30, 90), power = c(1/5, 1.5)))
# some_col_func <- colorspace::diverge_hcl
# some_col_func <- colorspace::sequential_hcl
some_col_func <- function(n) (colorspace::diverge_hcl(n, h = c(246, 40), c = 96, l = c(65, 90)))


# par(mar = c(3,3,3,3))
# library(gplots)
gplots::heatmap.2(as.matrix(animals-1), 
          main = "Attributes of Animals",
          srtCol = 35,
          Rowv = dend_r,
          Colv = dend_c,
          trace="row", hline = NA, tracecol = "darkgrey",         
          margins =c(6,3),      
          key.xlab = "no / yes",
          denscol = "grey",
          density.info = "density",
          col = some_col_func
         )
```

```
## NULL
```

We see that we have several groups of variables: the “can fly” and “endangered” (which usually are both “no”), the “have hair”, and the “warm-blooded”, “vertebrate”, and “live in groups”.

We see that within the animals there are (roughly!) the following 4 groups:

1. The cold-blooded non-vertebrates, which are mostly not endangered.
2. The warm-blooded vertebrates, which live in groups, have hair, cannot fly, and mostly are not endangered.
3. The cold-blooded vertebrates, without hair, cannot fly, and are not endangered.
4. The (mostly) warm-blooded vertebrates, without hair, some can fly, and some are endangered.

How much of a difference would we get if we used another clustering algorithm?

We first calculate the clustering using 8 different methods:

```
hclust_methods <- c("ward.D", "single", "complete", "average", "mcquitty", 
        "median", "centroid", "ward.D2")
animals_dendlist <- dendlist()

for(i in seq_along(hclust_methods)) {
   tmp_dend <-  animals %>% dist(method = "man") %>% 
      hclust(method = hclust_methods[i]) %>% as.dendrogram 
   animals_dendlist <- dendlist(animals_dendlist, tmp_dend)
}
names(animals_dendlist) <- hclust_methods
# votes.repub_dendlist
```

Next, we can look at the cophenetic correlation between each clustering result using `cor.dendlist` (This can be nicely plotted using the `corrplot` function from the *corrplot* package):

```
cophenetic_cors <- cor.dendlist(animals_dendlist)
corrplot::corrplot(cophenetic_cors, "pie", "lower")
```

We see that the different methods (other than ward.D and ward.D2), all give quite different results. So would the above analysis be different if we had used another clustering algorithm?

For this purpose, we compare the clustering solution of each algorithm with one another, when cut to k=4 clusters, using the Fowlkes-Mallows Index. This measure is similar to rand (or rand adjusted) index, and gives a value of 1 when the two clusters conform, and 0 when they do not:

```
remove_median <- dendlist(animals_dendlist, which = c(1:8)[-6] )
FM_cors <- cor.dendlist(remove_median, method = "FM_index", k = 4)
corrplot::corrplot(FM_cors, "pie", "lower")
```

We removed the “median” method since it did not have k=4 possible. In general, the results seems sensitive to the algorithm used, and the different algorithm methods do not seem to agree with one another (with regards to k=4), so further analyses may be in place in order to decide on which algorithm and interpretation are most appropriate for these data.

(Other possible data sets for the future: chorSub, flower, plantTraits, pluton, ruspini, agriculture)
