# Supplementary material for: dendextend: an R package for visualizing, adjusting and comparing trees of hierarchical clustering
Source: Bioinformatics. 2015 Jul 23;31(22):3718–20. doi: 10.1093/bioinformatics/btv428 (PMC4817050; doi:10.1093/bioinformatics/btv428)
Supplement: Supplementary Data [file supp_btv428_suppl_data.zip › Frequently asked questions.html]

Frequently asked questions


# Frequently asked questions

#### *Tal Galili*

#### *2015-07-17*

- Introduction
  - How to colour the labels of a dendrogram by an additional factor variable
  - How to color a dendrogram’s labels according to defined groups? (in R)
  - How to color a dendrogram’s branches/labels based on cluster (i.e.: cutree result)
  - Change dendrogram’s labels
  - Larger font for leaves in a dendrogram
  - How to view attributes of a dendrogram
  - How to color the branches in heatmap.2?
  - For package developers - how to call imported calls from dendextend 0.18.3?
  - How to plot a fan (Polar) Dendrogram in R?
  - A way to calculate lowest value of h in cut that produces groupings of a given minimum size?
  - Coloring dendrogram’s end branches (or leaves) based on column number of data frame in R

## Introduction

Questions are often taken from here the stackoverflow `dendrogram` tag.

### How to colour the labels of a dendrogram by an additional factor variable

Asked (http://stackoverflow.com/questions/27485549/how-to-colour-the-labels-of-a-dendrogram-by-an-additional-factor-variable-in-r)[here].

Solution: use the `labels_colors` function.

```
# install.packages("dendextend")
library(dendextend)

dend <- as.dendrogram(hclust(dist(USArrests[1:5,])))
# Like: 
# dend <- USArrests[1:5,] %>% dist %>% hclust %>% as.dendrogram

# By default, the dend has no colors to the labels
labels_colors(dend)
```

```
## NULL
```

```
par(mfrow = c(1,2))
plot(dend, main = "Original dend")

# let's add some color:
labels_colors(dend) <- 1:5
# Now each state has a color
labels_colors(dend)
```

```
##   Arkansas    Arizona California    Alabama     Alaska 
##          1          2          3          4          5
```

```
plot(dend, main = "A color for every state")
```

Instead of using `1:5`, we can obviously use colors that are based on another factor (organized): the labels themselves. But in such a case, we want to map between the order of the labels and the order of the items in the original dataset. Here is another example based on the iris dataset:

```
# install.packages("dendextend")
library(dendextend)

small_iris <- iris[c(1, 51, 101, 2, 52, 102), ]
dend <- as.dendrogram(hclust(dist(small_iris[,-5])))
# Like: 
# dend <- small_iris[,-5] %>% dist %>% hclust %>% as.dendrogram

# By default, the dend has no colors to the labels
labels_colors(dend)
```

```
## NULL
```

```
par(mfrow = c(1,2))
plot(dend, main = "Original dend")

# Let's add some color:
colors_to_use <- as.numeric(small_iris[,5])
colors_to_use
```

```
## [1] 1 2 3 1 2 3
```

```
# But sort them based on their order in dend:
colors_to_use <- colors_to_use[order.dendrogram(dend)]
colors_to_use
```

```
## [1] 1 1 2 2 3 3
```

```
# Now we can use them
labels_colors(dend) <- colors_to_use
# Now each state has a color
labels_colors(dend)
```

```
##   1   2  51  52 101 102 
##   1   1   2   2   3   3
```

```
plot(dend, main = "A color for every Species")
```

### How to color a dendrogram’s labels according to defined groups? (in R)

Asked (http://stackoverflow.com/questions/31117849/how-to-color-a-dendrograms-labels-according-to-defined-groups-in-r)[here].

Solution: use the `color_labels` function.

I suspect the function you are looking for is either `color_labels` or `get_leaves_branches_col`. The first color your labels based on `cutree` (like `color_branches` do) and the second allows you to get the colors of the branch of each leaf, and then use it to color the labels of the tree (if you use unusual methods for coloring the branches (as happens when using `branches_attr_by_labels`). For example:

```
 # define dendrogram object to play with:
 hc <- hclust(dist(USArrests[1:5,]), "ave")
 dend <- as.dendrogram(hc)

 library(dendextend)
 par(mfrow = c(1,2), mar = c(5,2,1,0))
 dend <- dend %>%
          color_branches(k = 3) %>%
          set("branches_lwd", c(2,1,2)) %>%
          set("branches_lty", c(1,2,1))
```

```
## Loading required namespace: colorspace
```

```
 plot(dend)

 dend <- color_labels(dend, k = 3)
 # The same as:
 # labels_colors(dend)  <- get_leaves_branches_col(dend)
 plot(dend)
```

Either way, you should always have a look at the `set` function, for ideas on what can be done to your dendrogram (this saves the hassle of remembering all the different functions names).

### How to color a dendrogram’s branches/labels based on cluster (i.e.: cutree result)

Use the `color_branches` and `color_labels` functions, with the `k` (or`h`) parameter:

```
# install.packages("dendextend")
library(dendextend)

dend <- as.dendrogram(hclust(dist(USArrests[1:5,])))
# Like: 
# dend <- USArrests[1:5,] %>% dist %>% hclust %>% as.dendrogram

dend1 <- color_branches(dend, k = 3)
dend2 <- color_labels(dend, k = 3)

par(mfrow = c(1,2))
plot(dend1, main = "Colored branches")
plot(dend2, main = "Colored labels")
```

### Change dendrogram’s labels

Use the left assign `labels<-` function:

```
# install.packages("dendextend")
library(dendextend)

dend <- as.dendrogram(hclust(dist(USArrests[1:5,])))
# Like: 
# dend <- USArrests[1:5,] %>% dist %>% hclust %>% as.dendrogram

labels(dend)
```

```
## [1] "Arkansas"   "Arizona"    "California" "Alabama"    "Alaska"
```

```
labels(dend) <- 1:5
labels(dend)
```

```
## [1] 1 2 3 4 5
```

### Larger font for leaves in a dendrogram

Asked (http://stackoverflow.com/questions/26965390/larger-font-and-spacing-between-leaves-in-r-dendrogram)[here].

Solution: use the `set` function, with the “labels\_cex” parameter.

```
# install.packages("dendextend")
library(dendextend)

dend <- as.dendrogram(hclust(dist(USArrests[1:5,])))
# Like: 
# dend <- USArrests[1:5,] %>% dist %>% hclust %>% as.dendrogram

# By default, the dend has no text size to it (showing only the first leaf)
get_leaves_nodePar(dend)[[1]]
```

```
## [1] NA
```

```
par(mfrow = c(1,2), mar = c(10,4,4,2))
plot(dend, main = "Original dend")

# let's increase the size of the labels:
dend <- set(dend, "labels_cex", 2)
# Now each state has a larger label
get_leaves_nodePar(dend)[[1]]
```

```
## $lab.cex
## [1] 2
## 
## $pch
## [1] NA
```

```
plot(dend, main = "A larger font for labels")
```

(Note that changing the spacing between the labels is currently not implemented)

### How to view attributes of a dendrogram

Asked (http://stackoverflow.com/questions/26240200/how-to-access-attributes-of-a-dendrogram-in-r)[here], and (http://stackoverflow.com/questions/25664911/r-hclust-height-of-final-merge)[here].

It generally depends on which attribute we want to view. For “midpoint” (or height) use the `get_nodes_attr` function, with the “midpoint” parameter.

```
# install.packages("dendextend")
library(dendextend)

dend <- as.dendrogram(hclust(dist(USArrests[1:5,])))
# Like: 
# dend <- USArrests[1:5,] %>% dist %>% hclust %>% as.dendrogram

# midpoint for all nodes
get_nodes_attr(dend, "midpoint")
```

```
## [1] 1.25   NA 1.50 0.50   NA   NA 0.50   NA   NA
```

```
# Perhaps also for the height:
get_nodes_attr(dend, "height")
```

```
## [1] 108.85192   0.00000  63.00833  23.19418   0.00000   0.00000  37.17701
## [8]   0.00000   0.00000
```

To also change an attribute, you can use the various assign functions from the package: `assign_values_to_leaves_nodePar`, `assign_values_to_leaves_edgePar`, `assign_values_to_nodes_nodePar`, `assign_values_to_branches_edgePar`, `remove_branches_edgePar`, `remove_nodes_nodePar`

### How to color the branches in heatmap.2?

Asked (http://stackoverflow.com/questions/29265536/how-to-color-the-branches-and-tick-labels-in-the-heatmap-2?)[here].

Solution: use the `color_branches` function (or the `set` function, with the “branches\_k\_color”, “k”, and “value” parameters).

(Getting the data for this example is from the (http://stackoverflow.com/questions/29265536/how-to-color-the-branches-and-tick-labels-in-the-heatmap-2)[original SO question])

```
test <- test0
rnames <- test[,1] 
test <- data.matrix(test[,2:ncol(test)]) # to matrix
rownames(test) <- rnames                 
test <- scale(test, center=T, scale=T) # data standarization
test <- t(test) # transpose


## Creating a color palette & color breaks

my_palette <- colorRampPalette(c("forestgreen", "yellow", "red"))(n = 299)

col_breaks = c(seq(-1,-0.5,length=100),  # forestgreen
               seq(-0.5,0.5,length=100), # yellow
               seq(0.5,1,length=100))    # red

# distance & hierarchical clustering
distance = dist(test, method ="euclidean")    
hcluster = hclust(distance, method ="ward.D")


dend1 <- as.dendrogram(hcluster)

# Get the dendextend package
if(!require(dendextend)) install.packages("dendextend")
library(dendextend)
# get some colors
cols_branches <- c("darkred", "forestgreen", "orange", "blue")
# Set the colors of 4 branches
dend1 <- color_branches(dend1, k = 4, col = cols_branches)
# or with:
# dend1 <- set(dend1, "branches_k_color", k = 4, value = cols_branches)

# Get the colors of the tips of the dendrogram:
# col_labels <- cols_branches[cutree(dend1, k = 4)] # this may need tweaking in various cases - the following is a more general solution.

col_labels <- get_leaves_branches_col(dend1)
# But due to the way heatmap.2 works - we need to fix it to be in the 
# order of the data!   
col_labels <- col_labels[order(order.dendrogram(dend1))]

dend1
# plot(dend1)
# a <- heights_per_k.dendrogram(dend1)
# library(dendextendRcpp)
# a2 <- heights_per_k.dendrogram(dend1)
# nleaves(dend1)


# Creating Heat Map
# if(!require(gplots)) install.packages("gplots")
library(gplots)
heatmap.2(test,  
    main = paste( "test"),  
        trace="none",          
        margins =c(5,7),      
        col=my_palette,        
        breaks=col_breaks,     
        dendrogram="row",      
        Rowv = dend1,  
        Colv = "NA", 
        key.xlab = "Concentration (index)",
        cexRow =0.6,
        cexCol = 0.8,
        na.rm = TRUE,
        RowSideColors = col_labels # to add nice colored strips     
      # colRow = col_labels # to add nice colored labels - only for qplots 2.17.0 and higher
        )
```

### For package developers - how to call imported calls from dendextend 0.18.3?

If you are developing a package and you wish to use dendextend as an imported package, that is - without loading it to the search path, you should run:

```
dendextend::assign_dendextend_options()
# This populates the dendextend::dendextend_options() space
```

Before using any of its function (for example: `dendextend::color_branches` ). As of dendextend version 1.0.0, this is no longer required.

### How to plot a fan (Polar) Dendrogram in R?

Asked (http://stats.stackexchange.com/questions/4062/how-to-plot-a-fan-polar-dendrogram-in-r)[here].

Solution: use the `circlize_dendrogram` function.

```
# install.packages("dendextend")
# install.packages("circlize")
library(dendextend)
library(circlize)

# create a dendrogram
hc <- hclust(dist(datasets::mtcars))
dend <- as.dendrogram(hc)

# modify the dendrogram to have some colors in the branches and labels
dend <- dend %>% 
   color_branches(k=4) %>% 
   color_labels

# plot the radial plot
par(mar = rep(0,4))
# circlize_dendrogram(dend, dend_track_height = 0.8) 
circlize_dendrogram(dend, labels_track_height = NA, dend_track_height = .4)
```

### A way to calculate lowest value of h in cut that produces groupings of a given minimum size?

Asked (http://stackoverflow.com/questions/31124810/r-cut-dendrogram-into-groups-with-minimum-size/)[here].

Solution: use the `heights_per_k.dendrogram` function.

This feature is available in the dendextend package with the `heights_per_k.dendrogram` function (which also has a faster C++ implementation when loading the dendextendRcpp function).

```
hc <- hclust(dist(USArrests[1:4,]), "ave")
dend <- as.dendrogram(hc)
heights_per_k.dendrogram(dend)
```

```
##        1        2        3        4 
## 86.47086 68.84745 45.98871 28.36531
```

As a sidenote, the dendextend package has a `cutree.dendrogram` S3 method for dendrograms (which works very similarly to `cutree` for `hclust` objects).

### Coloring dendrogram’s end branches (or leaves) based on column number of data frame in R

Asked (http://stackoverflow.com/questions/30062187/coloring-dendrogram-s-end-branches-or-leaves-based-on-column-number-of-data-fr)[here].

Solution: use the `assign_values_to_leaves_edgePar` function.

```
aa1 <- c(2,4,6,8)
bb1 <- c(1,3,7,11)
aa2 <- c(3,6,9,12)
bb2 <- c(3,5,7,9)
data.main <- data.frame(aa1,bb1,aa2,bb2)
d1 <- dist(t(data.main))
hcl1 <- hclust(d1)
# plot(hcl1)

dend <- as.dendrogram(hcl1)
col_aa_red <- ifelse(grepl("aa", labels(dend)), "red", "blue")
dend2 <- assign_values_to_leaves_edgePar(dend=dend, value = col_aa_red, edgePar = "col")
plot(dend2)
```
